# Supplementary material for: Effects of smoke flavoring using different wood chips and barbecuing on the formation of polycyclic aromatic hydrocarbons and heterocyclic aromatic amines in salmon fillets
Source: PLoS One. 2020 Jan 14;15(1):e0227508. doi: 10.1371/journal.pone.0227508 (PMC6959562; doi:10.1371/journal.pone.0227508)
Supplement: Attachment — Submitted filename: Response to reviewers.docx [file pone.0227508.s002.docx]

**Plos One**

PONE-D-19-23526R1

“**Effects of homemade smoking with different wood chips and barbecuing on the formation of polycyclic aromatic hydrocarbons and heterocyclic aromatic amines in salmon fillets**”

Dear Editor and Reviewers,

Thanks a bunch for your criticisms on our MS entitled “Effects of homemade smoking with different wood chips and barbecuing on the formation of polycyclic aromatic hydrocarbons and heterocyclic aromatic amines in salmon fillets”. We have taken all the comments into consideration. All amendments and corrections are highlighted yellow on the revised form of the MS. Herein with the answer point by point as listed below;

**Response to the reviewers:**

**Review Comments to the Author**

**Reviewer #1:**

**Research paper: "The effects of smoking with different wood chips and barbecuing on the formation of polycyclic aromatic hydrocarbons and heterocyclic aromatic amines in salmon" (PONE-D-19-23526_R1)

The author accepted most of the suggested corrections; still one change is necessary before the paper can be accepted for publication. Smoking is a technological process that includes salting, drying and smoke application. Please change homemade smoking to smoke flavoring throughout the text and update the text accordingly.**

Thank you for your valuable comment. The statement of “homemade smoking” has been changed as “smoke flavoring” throughout the manuscript.

**Title suggestion:**

**Effects of smoke flavoring using different wood chips and barbecuing on the formation of polycyclic aromatic hydrocarbons and heterocyclic aromatic amines in salmon fillets**

Thank you for your valuable suggestion. Title of the manuscript has been changed according to your suggestion.

The revised manuscript has been resubmitted to your journal. I look forward to your positive response as soon as possible. Is there anything I can do for that, please don’t hesitate to contact me.

Sincerely yours,

Dr. Emel Oz
